# Mapping of QLQ-C30 and QLQ-PR25 Scores to EQ-5D-5L Utility Values for Patients with Prostate Cancer Receiving Novel Androgen Receptor Signaling Inhibitors

**DOI:** 10.1016/j.euros.2025.12.005

**Published:** 2025-12-30

**Authors:** Shao-Yuan Hao, Shu-Pin Huang, Ching-Chia Li, Hung-Lung Ke, Kuang-Shun Chueh, Hsin-Chih Yeh, Hao-Wei Chen, Hsuan-Yu Hung, Chung-Yu Chen, Ichiro Arai

**Affiliations:** a Department of Pharmacy, Kaohsiung Medical University Hospital, Kaohsiung Medical University, Kaohsiung, Taiwan, ROC; b School of Pharmacy, Kaohsiung Medical University, Kaohsiung, Taiwan, ROC; c Department of Urology, Kaohsiung Medical University Hospital, Kaohsiung, Taiwan, ROC; d Graduate Institute of Clinical Medicine, College of Medicine, Kaohsiung Medical University, Kaohsiung, Taiwan, ROC; e Department of Urology, Kaohsiung Medical University Gangshan Hospital, Kaohsiung, Taiwan, ROC; f School of Post-Baccalaureate Medicine, College of Medicine, Kaohsiung Medical University, Kaohsiung, Taiwan, ROC; g Department of Urology, School of Medicine, College of Medicine, Kaohsiung Medical University, Kaohsiung, Taiwan, ROC; h Graduate Institute of Medicine, College of Medicine, Kaohsiung Medical University, Kaohsiung, Taiwan, ROC; i Department of Pharmacy, Ditmanson Medical Foundation Chia-Yi Christian Hospital, Chiayi, Taiwan, ROC; j Department of Medical Research, Kaohsiung Medical University Hospital, Kaohsiung, Taiwan, ROC; k Faculty of Pharmaceutical Sciences, Nihon Pharmaceutical University, Saitama, Japan

**Keywords:** EQ-5D-5L, Mapping, Prostatic neoplasms, QLQ-C30, QLQ-PR25, Quality of life

## Abstract

**Background and objective:**

Disease-specific questionnaires provide detailed health insights and are increasingly used in mapping studies to estimate utility values. Given the limited mapping studies in prostate cancer, our aim was to develop mapping algorithms to convert scores from disease-specific questionnaires to EQ-5D utility values for patients receiving novel androgen receptor signaling inhibitors (ARSIs).

**Methods:**

This cross-sectional study enrolled prostate cancer patients in Taiwan. Health-related quality of life was assessed using the EQ-5D-5L, European Organization for Research and Treatment of Cancer Quality of Life Questionnaire-Core 30-item (QLQ-C30), and QLQ-Prostate Cancer 25-item (QLQ-PR25) instruments. Mapping algorithms were developed using ordinary least squares (OLS) and Tobit regression. Model performance was evaluated using the mean absolute error, root mean square error, Akaike information criterion, and Bayesian information criterion.

**Key findings and limitations:**

A total of 100 patients were included. The mean EQ-5D index score was 0.71 (standard deviation 0.41), and the mean QLQ-C30 global health status score was 70 (standard deviation 21). Patients using an ARSI for ≥2 yr tended to report lower EQ-5D-5L index scores, global health status, and physical and social functioning, along with higher levels of fatigue, pain, and hormonal treatment–related symptoms. OLS and Tobit models demonstrated good predictive performance. However, given the small sample size, the results remain exploratory and are not intended for direct clinical use.

**Conclusions and clinical implications:**

This study provides real-world evidence on the HRQoL of Asian patients with prostate cancer on ARSI therapy. The results demonstrate that QLQ-C30 data can be effectively mapped to EQ-5D-5L utility values. These findings fill a gap in knowledge left by clinical trials.

**Patient summary:**

We tested whether quality of life scores reported by patients with prostate cancer who were taking a specific type of hormone therapy could be converted to values that are used in economic analyses. The results show that our mapping method can efficiently convert scores from the QLQ-C30 patient questionnaire to economic utility scores.

## Take Home Message

Patients with prostate cancer taking an androgen receptor signaling inhibitor (ARSI) for more than 2 yr experienced more fatigue, pain, and hormonal treatment–related symptoms. Mapping of QLQ-C30 and QLQ-PR25 scores to EQ-5D-5L utility values via ordinary least squares and Tobit regression methods is feasible for ARSI-treated patients.

## 1 Introduction

Prostate cancer is a common and slowly progressing malignancy in men, so long-term assessment of health-related quality of life (HRQoL) is essential [1], [2]. Treatments such as androgen deprivation therapy (ADT) and novel androgen receptor signaling inhibitors (ARSIs) can markedly affect daily life, but real-world HRQoL studies are limited [3], [4], [5], [6], [7], [8].

Cost-utility analysis requires quality-adjusted life years (QALYs) derived from utility values [9]. However, disease-specific instruments such as the European Organization for Research and Treatment of Cancer Quality of Life Questionnaire-Core 30-item (EORTC QLQ-C30) and the EORTC QLQ-Prostate Cancer 25-item (EORTC QLQ-PR25) for prostate cancer, lack the utility values needed for QALY estimation [10], [11]. The absence of generic patient-reported outcome measures (PROMs) such as the EuroQol five-dimension, five-level questionnaire (EQ-5D-5L) limits economic evaluation [12]. Mapping, which is a regression-based method that converts HRQoL scores to utility values, can bridge this gap [13].

In parallel, international initiatives such as the International Consortium for Health Outcomes Measurement (ICHOM) have developed standard sets for prostate cancer, and recommend both generic instruments (eg, EQ-5D) and disease-specific PROMs [14]. These efforts highlight the value of mapping as a mechanism for integrating widely collected disease-specific data into standardized economic evaluations.

To date, only one mapping study for patients with prostate cancer has been conducted in Western countries, raising concerns about its applicability to Asian populations [15]. The aim of our study was to present real-world HRQoL results and provide mapping algorithms from the QLQ-C30 (alone or with QLQ-PR25) to the EQ-5D-5L for patients with prostate cancer receiving ARSIs.

## 2 Patients and methods

### 2.1 Guidelines and ethical considerations

This study followed the methodological and reporting guidelines outlined in the MAPS (MApping onto Preference-based measures reporting Standards) statement [16]. Ethical approval was obtained from the institutional ethics committee of Kaohsiung Medical University Hospital (KMUHIRB-E(I)-20200070). All procedures were conducted in compliance with the Declaration of Helsinki.

### 2.2 Study population

This cross-sectional study was conducted at a medical center and a regional hospital in southern Taiwan. We included patients receiving a novel ARSI (abiraterone, enzalutamide, apalutamide, or darolutamide) for at least 28 d, and excluded patients aged <20 yr and those with other cancers or with cognitive dysfunction. Details of the sample size estimation are presented in the Supplementary material [17].

### 2.3 Data collection

Data collection involved face-to-face interviews to gather HRQoL data, personal characteristics, disease information, and medical history. Operational definitions are detailed in Supplementary Table 1, and the index date was set as the questionnaire completion date.

### 2.4 HRQoL instruments

The HRQoL instruments used in the study were the Taiwan version of the EQ-5D-5L, QLQ-C30, and QLQ-PR25 questionnaires [10], [11], [12]. The EQ-5D-5L is a generic HRQoL tool with five dimensions (mobility, self-care, usual activities, pain/discomfort, and anxiety/depression), each rated on a five-level scale; an index score can be calculated on the basis of each country’s value set [18]. A higher index score indicates better QoL. The EQ-5D-5L also includes a Visual Analog Scale from 0 to 100, for which higher scores indicate better QoL. The QLQ-C30 has 30 items, including a global health status scale, five functional scales (physical, role, emotional, cognitive, and social), and nine symptom scales (fatigue, nausea/vomiting, pain, dyspnea, insomnia, appetite loss, constipation, diarrhea, and financial difficulties). The QLQ-PR25 has 25 items, with two functional scales (sexual activity/functioning) and four symptom scales (urinary, bowel, hormonal treatment–related symptoms, and incontinence aids). Higher QLQ scores for global health status and functional scales denote better QoL, while higher scores for symptom scales indicate worse QoL [19].

### 2.5 Population applicability and conceptual overlap

The applicability of the questionnaires to the Taiwanese population was confirmed by previous validation studies [20], [21]. Previous oncology studies have also demonstrated sufficient conceptual overlap by mapping the QLQ-C30 to the EQ-5D [22], [23].

### 2.6 Statistical analysis

#### 2.6.1 HRQoL outcomes

EQ-5D-5L index scores were derived using the Taiwan hybrid model [24]. QLQ-C30 and QLQ-PR25 were scored according to the EORTC scoring manual [25]. Descriptive results for HRQoL subgroups are presented by ARSI agent (abiraterone, enzalutamide, apalutamide, darolutamide), ARSI duration (<2 yr vs ≥2 yr), and metastatic status. We also explored associations between EQ-5D-5L index scores and demographics using descriptive univariable and multivariable analyses. Variable selection was based on prior research rather than statistical significance [26], [27], [28], [29].

Statistical results for continuous variables are purely descriptive. We assessed between-group differences in EQ-5D-5L index scores and QLQ-C30 subscales against published minimally important differences (Supplementary Table 2). In line with the descriptive approach, a two-sided *p* value <0.05 was treated as exploratory only.

#### 2.6.2 Mapping algorithms

For construction of the mapping models, we first confirmed that there was sufficient correlation between EQ-5D-5L and the QLQ-C30/QLQ-PR25 subscales. Correlations among QLQ-C30 and QLQ-PR25 domains were also examined, and interaction terms were considered only when correlations met the predefined threshold (criteria in Supplementary Table 3). Following confirmation of sufficient correlation, both ordinary least squares (OLS) and Tobit regression models were developed for mapping. OLS served as the primary model for reporting results owing to its robust predictive performance, while Tobit regression was additionally applied as a sensitivity analysis to account for the ceiling effect of EQ-5D-5L scores and to examine the consistency of estimates under a censored distribution [13], [30]. To enhance the prediction accuracy, we first analyzed the distribution of EQ-5D-5L index scores. This was to determine if an alternative regression method, such as the adjusted limited dependent variable mixture model (ALDVMM), was needed to handle a multicomponent distribution [31].

The EQ-5D index score served as the dependent variable for the eight model specifications, which are detailed in Supplementary Table 4. All QLQ-C30 and QLQ-PR25 subscales were included as candidate predictors, except for sexual functioning and incontinence aids, for which some patients could not be scored because of the scoring rules of the instrument. The sexual activity subscale was retained to reflect the impact of sexual function on QoL. Age was included as an additional variable in model 4 and model 8 because it is a well-established determinant of HRQoL and may capture unmeasured factors not reflected in the QLQ-C30 or QLQ-PR25 items. This was prespecified on the basis of previous studies [22], [23], [32].

Model performance was evaluated using the mean absolute error (MAE), root mean square error (RMSE), Akaike information criterion (AIC), Bayesian information criterion (BIC), adjusted R^2^ value, and the log-likelihood ratio. Predictive stability was verified via fivefold cross-validation. The F test and likelihood ratio test were used to compare the fit between models. Data analysis was performed using SAS version 9.4 (SAS Institute, Cary, NC, USA) and R version 4.2.2 (R Foundation for Statistical Computing, Vienna, Austria).

## 3 Results

### 3.1 Study participants

**Fig. 1:**
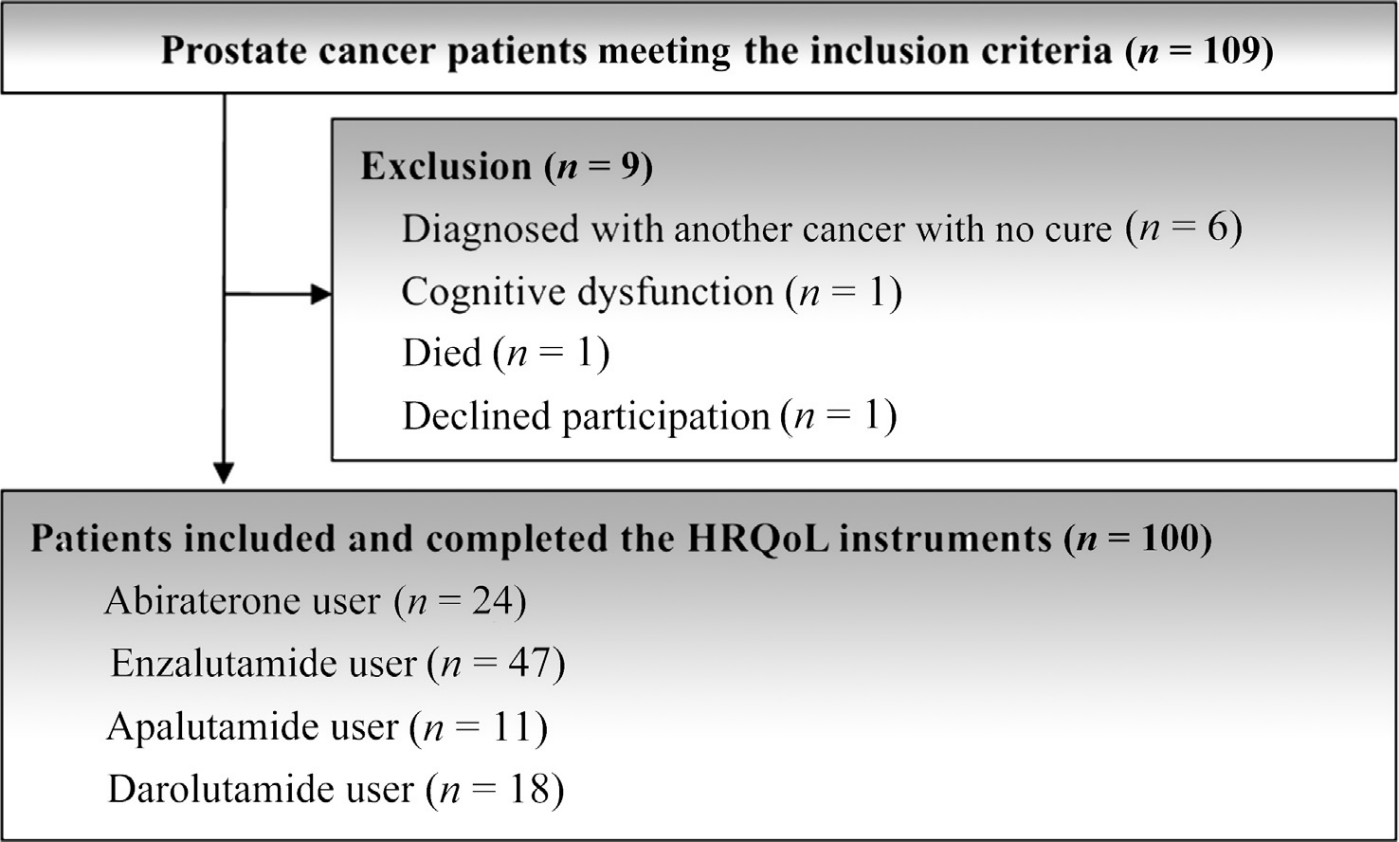
Study flow diagram. HRQoL = health-related quality of life.

Between September 2022 and August 2024, 109 Taiwanese patients with prostate cancer met the eligibility criteria. Ultimately, 100 patients on ARSI therapy completed the three HRQoL questionnaires (Fig. 1). The participant characteristics are list in Table 1.

**Table 1:** Characteristics of the study population (*n* = 100)

Parameter	Result
Median age, yr, (IQR)	75 (69–81)
Age group, yr, *n* (%)	
<60	4 (4)
60–69	28 (28)
70–79	40 (40)
≥80	28 (28)
Median body mass index, kg/m^2^ (IQR)	25 (23–27)
Employment status, *n* (%)	
Full time	9 (9)
Part-time	4 (4)
Retired	85 (85)
Data missing	2 (2)
Marital status, *n* (%)	
Single	5 (5)
Married	84 (84)
Widowed	11 (11)
Living arrangements, *n* (%)	
Live alone	8 (8)
Live with family	92 (92)
Wheelchair user, *n* (%)	15 (15)
Diaper use, *n* (%)	32 (32)
Median PSA at index date, ng/ml (IQR)	0.40 (0.03–2.5)
Metastasis stage, *n* (%)	
M0	18 (18)
M1	82 (82)
Castration response, *n* (%)	
Nonmetastatic castration-sensitive PC	1 (1)
Nonmetastatic castration-resistant PC	17 (17)
Metastatic castration-sensitive PC	52 (52)
Metastatic castration-resistant PC	30 (30)
Tumor metastasis site, *n* (%)	
Bone	77 (77)
Lymph node	17 (17)
Lung	5 (5)
Other organs	2 (2)
Median disease duration, yr (IQR)	2.6 (1.1–5.7)
Clinical stage, *n* (%)	
I	1 (1)
II	3 (3)
III	10 (10)
IV	84 (84)
Data missing	2 (2)
Prior treatment, *n* (%)	
Radical prostatectomy	36 (36)
Radiation therapy	29 (29)
ADT	100 (100)
Chemotherapy	9 (9)
Prior ADT, n (%)	
LHRH agonist	89 (89)
LHRH antagonist	30 (30)
Estrogen analog	7 (7)
Bicalutamide	57 (57)
ARSI	6 (6)
Current ADT, *n* (%)	
LHRH agonist	86 (86)
LHRH antagonist	14 (14)
Estrogen analog	1 (1)
Current ARSI, *n* (%)	
Abiraterone	24 (24)
Enzalutamide	47 (47)
Apalutamide	11 (11)
Darolutamide	18 (18)
Median ARSI duration, mo (IQR)	7.5 (3.8–19)
ARSI duration category (*n*)	
<6 mo	40
6–12 mo	25
12–18 mo	9
18–24 mo	13
>24 mo	13
Comorbidity, *n* (%)	
Cardiovascular disease	37 (37)
Hypertension	66 (66)
Diabetes	32 (32)
Hyperlipidemia	43 (43)
Chronic kidney disease	15 (15)
Liver disease	17 (17)
Respiratory disease	7 (7)
Depression	5 (5)
Dental illness	12 (12)
Orthopedics disease	22 (22)
Urinary medications	69 (69)
Gout	13 (13)

ADT = androgen deprivation therapy; ARSI = androgen receptor signaling inhibitor; LHRH = luteinizing hormone–releasing hormone; PC = prostate cancer; PSA = prostate-specific antigen.

### 3.2 Summary of questionnaire data and latent constructions

#### 3.2.1 EQ-5D-5L, QLQ-C30, and QLQ-PR25 scores

Across all five EQ-5D-5L dimensions, the majority of patients reported no problems (level 1), and no patients reported the most severe level (level 5) for the anxiety/depression dimension. For QLQ-PR25, only urinary symptoms and incontinence aids had a mean score >20 (Table 2).

**Table 2:** Health-related quality-of-life scores for the study population (*n* = 100)

Instrument and domain	Result
EQ-5D-5L	
Mean EQ-5D index score (SD)	0.71 (0.41)
Median EQ-5D index score (IQR)	0.87 (0.65–1)
EQ-5D index score range	−0.67 to 1
Patients with full health (EQ-5D index = 1), *n* (%)	29 (29)
Mobility level 1/2/3/4/5 (*n*)	55/22/12/1/10
Self-care level 1/2/3/4/5 (*n*)	81/5/5/2/7
Usual activities level 1/2/3/4/5 (*n*)	71/14/8/3/4
Pain/discomfort level 1/2/3/4/5 (*n*)	49/36/8/5/2
Anxiety/depression level 1/2/3/4/5 (*n*)	62/28/7/3/0
Mean EQ VAS score (SD)	74 (18)
Mean QLQ-C30 scores (SD)	
Global health status	70 (21)
Physical functioning	72 (29)
Role functioning	77 (32)
Emotion functioning	84 (19)
Cognitive functioning	81 (20)
Social functioning	81 (25)
Fatigue	31 (28)
Nausea/vomiting	4.5 (11)
Pain	22 (27)
Dyspnea	19 (26)
Insomnia	27 (30)
Appetite loss	14 (24)
Constipation	18 (28)
Diarrhea	8.3 (17)
Financial difficulties	12 (20)
Mean QLQ-PR25 scores (SD)	
Sexual activity	1.3 (4.5)
Sexual functioning	–
Urinary symptoms	25 (18)
Bowel symptoms	8.7 (11)
Hormonal treatment–related symptoms	13 (11)
Incontinence aids (*n* = 32)	23 (33)

EQ-5D-5L = EuroQol five-dimension five-level questionnaire; EQVAS = EuroQol Visual Analog Scale; QLQ-C30 = Quality of Life Questionnaire-Core 30-item; QLQ-PR25 = Quality of Life Questionnaire-Prostate Cancer 25-item; SD = standard deviation.

Scores for physical, role, and social functioning and pain varied by ARSI agent (Supplementary Tables 5). Prolonged ARSI treatment (≥2 yr) was associated with declines in the EQ-5D-5L index score and scores for global health status and physical and social functioning, and with increased fatigue, pain, and hormonal treatment–related symptoms (Supplementary Table 6). Patients with nonmetastatic prostate cancer tended to have better physical, role, and social functioning than patients with metastatic disease (Supplementary Table 7).

#### 3.2.2 Demographic factors associated with EQ-5D-5L index scores

Exploratory univariable and multivariable regression analyses were conducted to examine potential associations between demographic variables and EQ-5D-5L index scores. Exploratory multivariable analysis revealed that lower EQ-5D-5L index scores were associated with higher prostate-specific antigen at the index date (estimate −0.003, 95% CI −0.005 to −0.0001; *p* = 0.04) and with wheelchair use (estimate −0.64, 95% CI −0.86 to −0.42; *p* < 0.001; Supplementary Table 8).

### 3.3 Mapping results

The EQ-5D index score showed sufficient correlation with the QLQ-C30/QLQ-PR25 subscales (Supplementary Table 9). Intercorrelations between these subscales were also confirmed to determine the appropriate interaction terms for inclusion in the models (Supplementary Table 10). We evaluated eight mapping models. The performance results for OLS models 1–8 are presented in Supplementary Table 11.

On the basis of the average ranking for the MAE, RMSE, AIC, and BIC metrics, model 8 (which includes QLQ-C30 subscales, squared terms, interaction terms, and age) had the best overall performance. Model 4, which includes the QLQ-PR25 subscales in addition to the parameters in model 8, ranked second.

For the sensitivity analysis, performance results for Tobit models 1–8 are presented in Supplementary Table 12. On the basis of the average ranking for the metrics, model 7 (which includes QLQ-C30 subscales, squared terms, and interaction terms) was identified as the best-performing model with Tobit regression. Model 8 was ranked second, which differs from model 7 in the addition of age.

Tobit regression revealed a trend towards less overestimation at the lower end and less underestimation at the upper end of predicted utilities in comparison to OLS regression (Supplementary Table 13). The OLS F test and the likelihood ratio test for Tobit regression were used to compare the fit between models. The results indicate that inclusion of QLQ-PR25 subscales in the models provided no statistically significant improvement (Supplementary Tables 14 and 15).

## 4 Discussion

### 4.1 Real-world HRQoL insights

This is the first study to use EQ-5D-5L, QLQ-C30, and QLQ-PR25 to comprehensively assess the HRQoL of patients receiving novel ARSIs. In terms of the characteristics of prostate cancer populations in previous studies, the median age at diagnosis was 71 yr (IQR 66–77) in a multicenter, prospective observational study by Lim et al. [33] involving four Asian cohorts (Hong Kong, China, Malaysia, and Taiwan). In a real-world study involving patients with prostate cancer from the USA, five European countries, and Japan, the mean age was 72 yr (standard deviation 8.0) [34]. In a cross-sectional study by Karaihira et al. [35] involving 62 patients with prostate cancer in a Kenyan tertiary health facility, the mean age was 71 yr (standard deviation 7.4). The median patient age in our cohort of 75 yr is slightly higher than in these studies. A possible reason may be that most patients (84%) in our cohort had stage IV disease.

In addition, more patients received LHRH agonists than antagonists in our study, which aligns with findings from other studies [33], [35]. Consistent with other studies, bone was identified as the primary site of metastasis, followed by the lymph nodes, lungs, and liver [34].

The median EQ-5D index score for our ARSI cohort was 0.87, which is comparable to the range of 0.77–1 reported by Lim et al. [33] for Asian patients undergoing ADT.

While some individuals believe that sexual dysfunction does not impact their masculinity, international studies suggest that cultural differences link post-treatment erectile dysfunction to a reduced sense of masculinity. Given the highly individual nature of these perceptions, discussion of specific contributory factors with each patient is crucial for better HRQoL support [36].

### 4.2 Mapping

As the first study to construct mapping algorithms using the EQ-5D-5L, QLQ-C30, and QLQ-PR25 for patients on novel ARSIs, we excluded the ALDVMM mapping method because of the small sample size and the lack of a multicomponent distribution in the EQ-5D-5L index scores [22], [37]. OLS and Tobit models were sufficient for mapping. The results were consistent across both models, which supports the robustness of the mapping algorithm. As demographic information reflects individual differences that may significantly influence HRQoL scores, studies have incorporated parameters such as sex and age as independent variables in mapping model analysis [22], [23]. Age was also included in some of our mapping models (model 4 and model 8). Under the OLS approach, models that included age ranked highest in overall performance in comparison to those without age, although the differences in actual predicted values were minimal. In the sensitivity analysis using Tobit regression, addition of age did not lead to improvement in model ranking. This result leads us to suggest that multiple versions of mapping models, both including and excluding demographic variables, should be constructed to address generalizability across different populations. In addition, some studies include varying disease severities in mapping models [38]. However, the low heterogeneity of our population (84% clinical stage IV) led us to exclude different clinical stages from the regression model. Furthermore, we found that incorporation of squared and interaction terms modestly enhanced the predictive performance, which suggests that accounting for potential nonlinear effects between continuous predictors and utility outcomes improve the mapping accuracy. This is consistent with previous studies [22], [39].

Regarding the HRQoL instruments used, a mapping study by Wu et al. [15] in prostate cancer adopted a similar approach, though the authors combined the QLQ-C30 and FACT-P questionnaires. Our study differs from that of Wu et al. in both patient population and model variables. We focused on Taiwanese patients with prostate cancer taking ARSIs, while We et al. included patients from North America, Europe, and Australia with metastatic hormone-refractory prostate cancer. Our best-fit model incorporated QLQ-C30 subscales, whereas the model used by Wu et al. [15] comprised FACT-P subscales. Owing to the hormone-related impact of ARSIs on urinary and sexual function, we selected QLQ-PR25 over FACT-P, as the former is more focused on these issues. However, our study demonstrated that incorporation of QLQ-C30 subscales alone could accurately predict the EQ-5D index score without the need for QLQ-PR25. This finding aligns with other mapping research, such as a study by Proskorovsky et al. [40] that also demonstrated the strong predictive power of the QLQ-C30 alone.

As for predictive ability, our study confirmed that the OLS models were susceptible to over- and under-prediction of the EQ-5D-5L index score, consistent with previous mapping research [22], [38]. This issue was less evident in the Tobit models.

Our study has several limitations. First, as a cross-sectional study, it cannot establish cause-and-effect relationships, so the associations observed should not be interpreted as causal. Second, recall bias may have affected self-reported HRQoL, with potential for overestimation or underestimation of symptom severity. Third, selection bias is possible because inpatients and severely frail patients were excluded, which may have led to overestimation of HRQoL. In addition, the analyses of demographic factors associated with EQ-5D-5L were exploratory and based on prior evidence of their relevance to HRQoL with the aim of identifying potential predictors for future hypothesis testing rather than establishing definitive associations. These findings may not be replicated in external data sets. Furthermore, given the exploratory nature of the study, the mapping algorithms are not yet intended for direct clinical use, but may serve as a preliminary tool for research and health economic analyses when EQ-5D-5L data are unavailable. Finally, the relatively small sample size may limit the generalizability and increase the risk of model overfitting.

Despite its limitations, our study provides real-world evidence on the HRQoL of patients using ARSIs. The results also offer preliminary support for the development of mapping algorithms to predict EQ-5D scores in this population. Further research with larger, more diverse, and prospective cohorts is needed to validate these findings and improve the generalizability of the mapping algorithms.

## 5 Conclusions

We comprehensively evaluated HRQoL in a cohort of patients with prostate cancer on ARSIs and created preliminary mapping models from QLQ-C30 (alone or with QLQ-PR25) to EQ-5D-5L. The findings are exploratory and not intended for direct clinical application. Rather, our results could facilitate health economic evaluations or retrospective studies when EQ-5D data are not available. External validation in larger cohorts is required before clinical implementation.

  ***Author contributions***: Chung-Yu Chen has full access to all the data in the study and takes responsibility for the integrity of the data and the accuracy of the data analysis.

  *Study concept and design*: Hao, Huang, Li, C.-Y. Chen.

*Acquisition of data*: Hao, Huang, Li, Ke, Chueh, Yeh, H.-W. Chen, C.-Y. Chen.

*Analysis and interpretation of data*: Hao, C.-Y. Chen.

*Drafting of the manuscript*: Hao, Hung, C.-Y. Chen, Arai.

*Critical revision of the manuscript for important intellectual content*: Hao, Huang, Li, Ke, Chueh, Yeh, H.-W. Chen, Hung, C.-Y. Chen, Arai.

*Statistical analysis*: Hao, C.-Y. Chen.

*Obtaining funding*: C.-Y. Chen.

*Administrative, technical, or material support*: Hung, C.-Y. Chen.

*Supervision*: Huang, Li, C.-Y. Chen, Arai.

*Other*: None.

  ***Financial disclosures:*** Chung-Yu Chen certifies that all conflicts of interest, including specific financial interests and relationships and affiliations relevant to the subject matter or materials discussed in the manuscript (eg, employment/affiliation, grants or funding, consultancies, honoraria, stock ownership or options, expert testimony, royalties, or patents filed, received, or pending), are the following: None.

  ***Funding/Support and role of the sponsor*:** This work was supported by a grant from Kaohsiung Medical University (KMU-S114008). The sponsor played a role in management of the data.
