# Supplementary material for: Mapping of QLQ-C30 and QLQ-PR25 Scores to EQ-5D-5L Utility Values for Patients with Prostate Cancer Receiving Novel Androgen Receptor Signaling Inhibitors
Source: Eur Urol Open Sci. 2025 Dec 30;83:219–25. doi: 10.1016/j.euros.2025.12.005 (PMC12803998; doi:10.1016/j.euros.2025.12.005)
Supplement: Supplementary Data 1 [file mmc1.pdf]

Mapping of QLQ-C30 and QLQ-PR25 Scores to EQ-5D-5L Utility Values for Patients with Prostate Cancer Receiving Novel Androgen Receptor Signaling Inhibitors

Supplementary Materials

Table of Contents

Section 1 Supplementary data of methods

Section 2 Supplementary data on health-related quality of life

Section 3 Supplementary data on mapping

Section 1 Supplementary data of methods

1. Sample size estimation

(1) The sample size for the health-related quality of life questionnaires

The study aimed to investigate the health-related quality of life (HRQoL) among prostate cancer patients treated with androgen receptor signaling inhibitors (ARSIs) at two hospitals. The target population size was estimated to be approximately 120 patients, from which the Krejcie and Morgan formula determined a required sample size of 95 [1]. The Krejcie and Morgan formula is as follows:

$$n = \frac{\mathrm{N}X^2P(1-P)}{d^2(N-1)+X^2P(1-P)}$$

n = estimated sample size

N = population size

X = the table value of chi-square for 1 degree of freedom at desired confidence level (X = 1.96, X^2 = 3.84)

P = expected proportion (assumed 0.5 to estimate the maximum sample size)

d = the degree of accuracy expressed as a proportion (0.05)

The required sample size for this study

= (120*3.8*0.5*0.5) / 0.05^2(120-1) + 3.8*0.5*0.5

= 114/1.2

≈ 95

(2) The sample size for the mapping models

Although the sample size was calculated based on the Krejcie and Morgan formula to ensure adequate representativeness for the HRQoL survey, this estimation did not consider the number of predictors or model complexity required for multivariable mapping. As such, the mapping analyses were exploratory and may be prone to overfitting due to the limited sample size. External validation in larger, independent cohorts is warranted to improve generalizability and predictive robustness. However, given the relatively small population of patients receiving ARSIs in real-world practice, this study has already captured the maximum feasible number of participants available at the study sites.

Table s1 - Operational definitions of demographic items

Demographic items	Definitions
Age (year)	calculated as the index date minus the date of birth
BMI	calculated using weight and height from within six months prior to the index date
Occupation status	full time, part-time, and retired
Marital status	single, married, and widowed
Habitation	living alone or living with family
Wheelchair using	use wheelchair at index date
With diaper	use diaper at index date
PSA at index date (ng/mL)	collected from within six months prior to index date
Metastasis stage	defined by Union for International Cancer Control (UICC) 8th edition
Tumor metastasis	regions of metastasis were classified as bone, lymph node, lung, and other organs
Disease duration (year)	defined as the time from prostate cancer diagnosis to index date
Clinical staging	defined by Union for International Cancer Control (UICC) 8th edition
Prior treatment	treatment for prostate cancer classified as radical prostatectomy, radiation therapy, ADT, and chemotherapy
Prior ADT	classified as LHRH agonist, LHRH antagonist, estrogen analog, bicalutamide, novel ARSI
Current ADT	classified as LHRH agonist, LHRH antagonist, estrogen analog
Novel ARSI	abiraterone, enzalutamide, apalutamide, and darolutamide
Comorbidity	
Cardiovascular disease	two diagnostic records or prescriptions over 28 days
Hypertension	two diagnostic records or prescriptions over 28 days
Diabetes	two diagnostic records or prescriptions over 28 days
Hyperlipidemia	two diagnostic records or prescriptions over 28 days
Chronic kidney disease	two diagnostic records
Liver disease	two diagnostic records
Respiratory disease	two diagnostic records or prescriptions over 28 days
Depression	two diagnostic records or prescriptions over 28 days
Dental illness	two diagnostic records or prescriptions over 28 days
Orthopedics disease	two diagnostic records or prescriptions over 28 days
With urinary medications	two diagnostic records or prescriptions over 28 days
Gout	two diagnostic records or prescriptions over 28 days

ADT = androgen deprivation therapy; ARSI = androgen receptor signaling inhibitor; BMI = body mass index; LHRH = luteinizing-hormone releasing hormone; PSA = prostate-specific antigen.

Table s2 - Anchor-based and distribution-based MIDs for QLQ-C30 scales

Scale	Anchor-based		Distribution-based
	MID between groups		0.3 SD
	Improvement	Deterioration	
Global health status	No MID	-6	5.5
Physical functioning	No MID	-7	4.8
Role functioning	5	-10	5.6
Social functioning	3	-4	4.7
Fatigue	No MID	-7	5.6
Pain	No MID	-5	5.4
Diarrhea	13	-9	4.5

MID = minimally important differences; SD = standard deviation.

0.3 SD: The 0.3 SD approach is a distribution-based method for estimating the MID, indicating that a mean score changes exceeding 30% of the standard deviation may have clinical significance. It is one of the most used and conservative criteria in HRQoL research.

In this section, between-group differences in EQ-5D-5L index scores and QLQ-C30 subscales were evaluated in reference to published minimally important differences (MIDs).

According to instrument-defined estimates based on the Taiwan EQ-5D-5L value set, the MID was approximately **0.10** (95% CI: 0.098–0.10) [2].

For QLQ-C30, we adopted anchor-based and distribution-based MID for between-group improvement or deterioration as reported in previous validation studies [3]. To avoid overestimating clinical significance, the most conservative estimates (i.e., the largest MID thresholds reported) were adopted as references.

Table s3 - Correlation definitions

Correlation	Spearman's correlation coefficient (ρ)
Strong	$\rho \geq 0.6$
Moderate	$0.4 \leq \rho < 0.6$
Weak	$\rho \leq 0.4$

ρ = Spearman's correlation coefficients.

Spearman's correlation was considered sufficient when it reached a moderate to high level. When building the mapping model, we examined the intercorrelation results between the QLQ-C30 and QLQ-PR25 subscales. Interaction terms with correlation coefficients greater than 0.6 (strong) were included in the mapping model.

Table s4 - Model specifications of mapping

Model	Independent variables (X)
Model 1	QLQ-C30 global health status + QLQ-C30 subscales + QLQ-PR25 subscales
Model 2	QLQ-C30 global health status + QLQ-C30 subscales + QLQ-PR25 subscales + squared terms
Model 3	QLQ-C30 global health status + QLQ-C30 subscales + QLQ-PR25 subscales + squared terms + interaction terms
Model 4	QLQ-C30 global health status + QLQ-C30 subscales + QLQ-PR25 subscales + squared terms + interaction terms + age
Model 5	QLQ-C30 global health status + QLQ-C30 subscales
Model 6	QLQ-C30 global health status + QLQ-C30 subscales + squared terms
Model 7	QLQ-C30 global health status + QLQ-C30 subscales + squared terms + interaction terms
Model 8	QLQ-C30 global health status + QLQ-C30 subscales + squared terms + interaction terms + age

QLQ-C30 = Quality of Life Questionnaire Core 30-item; QLQ-PR25 = Quality of Life Questionnaire Prostate Cancer 25-item.

Note:

1. The input variables included all QLQ-C30 and QLQ-PR25 subscales except “sexual functioning” and “incontinence aid”, as not all patients received scores on them. The missing data in the “sexual functioning” and “incontinence aid” subscales are attributable to the scoring structure of the QLQ-PR25 questionnaire (please refer to Table 2 for the subscales of the QLQ-PR25). No patients reported engaging in sexual intercourse during the past four weeks; therefore, the sexual functioning domain could not be scored. Similarly, the “incontinence aid” item evaluates the patient’s perceived impact of using incontinence pads or diapers. Patients who did not use such aids were not assigned a score for this item. Thus, the missing values do not reflect non-response or refusal to answer but are inherent to the questionnaire design.
2. Non-linear modeling was addressed by incorporating **squared terms of QLQ-C30 and QLQ-PR25 subscales** in Models 2, 3, 4, 6, 7, and 8, with interaction terms (Models 3, 4, 7, and 8) added to capture potential interactive relationships.

Section 2 Supplementary data on health-related quality of life

Table s5 - HRQoL among the four ARSI groups

HRQoL	abiraterone (n = 24)	enzalutamide (n = 47)	apalutamide (n = 11)	darolutamide (n = 18)
EQ-5D-5L				
EQ-5D index score [a]	0.68 (0.43)	0.71 (0.39)	0.57 (0.48)	0.82 (0.39)
EQ VAS	79 (16)	69 (19)	76 (19)	80 (16)
QLQ-C30				
Global health status	72 (19)	67 (21)	72 (28)	72 (21)
Physical functioning [b]	72 (29)	70 (30)	70 (31)	78 (29)
Role functioning [b]	77 (32)	75 (34)	73 (32)	85 (30)
Emotion functioning	87 (15)	86 (19)	69 (27)	87 (15)
Cognitive functioning	79 (17)	84 (17)	74 (35)	80 (19)
Social functioning [c]	80 (24)	80 (26)	73 (28)	91 (17)
Fatigue	31 (26)	31 (29)	36 (33)	29 (27)
Nausea/Vomiting	4.9 (10)	4.3 (11)	7.6 (16)	2.8 (6.4)
Pain [d]	30 (28)	17 (23)	33 (27)	19 (32)
Dyspnea	22 (27)	19 (25)	18 (17)	13 (33)
Insomnia	28 (29)	25 (27)	36 (35)	26 (33)
Appetite loss	14 (17)	15 (28)	21 (31)	7.4 (18)
Constipation	15 (26)	15 (25)	33 (39)	20 (31)
Diarrhea	6.9 (14)	7.8 (16)	9.1 (16)	11 (26)
Financial difficulties	13 (22)	12 (18)	12 (17)	9.3 (25)
QLQ-PR25				
Sexual activity	0.69 (3.4)	0.35 (2.4)	3.0 (6.7)	3.7 (7.1)
Sexual functioning	-	-	-	-
Urinary symptoms	24 (19)	25 (18)	33 (25)	23 (13)
Bowel symptoms	5.9 (9.0)	8.5 (9.1)	15 (17)	8.8 (12)
Hormonal treatment-related symptoms	15 (13)	12 (9.4)	16 (15)	9.9 (8.0)
Incontinence aid	14 (26) [n = 7]	22 (37) [n = 15]	40 (44) [n = 5]	20 (18) [n = 5]

ARSI = androgen receptor signaling inhibitors; EQ-5D-5L = EuroQoL five-dimension five-level questionnaire; EQVAS = EuroQoL visual analogue scale; HRQoL = health-related quality of life; QLQ-C30 = Quality of Life Questionnaire Core 30-item; QLQ-PR25 = Quality of Life Questionnaire Prostate Cancer 25-item.

Assessment of whether the mean difference between groups exceeds the MID threshold (Table s5):

[a] Pairwise comparisons showed that darolutamide was associated with higher EQ-5D-5L index scores compared with apalutamide, abiraterone, and enzalutamide, all exceeding the reference MID. Conversely, apalutamide had lower scores compared with abiraterone and enzalutamide, also exceeding the MID threshold.

[b] Pairwise comparisons indicated that darolutamide resulted in better condition in physical and role functioning than enzalutamide and apalutamide, both exceeding the MID threshold, suggesting potential clinical relevance.

[c] Pairwise comparisons indicated that darolutamide resulted in better condition in social functioning than apalutamide, abiraterone, and enzalutamide, all exceeding the MID threshold. Additionally, enzalutamide and abiraterone had a better condition in social functioning than apalutamide, also exceeding the MID threshold.

[d] Pairwise comparisons indicated that enzalutamide and darolutamide were associated with lower pain scores compared with abiraterone, both exceeding the MID threshold. Conversely, apalutamide showed higher pain scores than enzalutamide and darolutamide, also exceeding the MID threshold, suggesting clinically meaningful difference

Table s6 - HRQoL of patients with ARSI duration over and under 2 years

HRQoL	ARSI duration < 2 years (n = 87)	ARSI duration≥2 years (n = 13)
EQ-5D-5L		
EQ-5D index score *	0.74 (0.36)	0.48 (0.63)
EQ VAS	74 (18)	73 (22)
QLQ-C30		
Global health status *	71 (21)	64 (24)
Physical functioning *	74 (28)	57 (36)
Role functioning	78 (31)	71 (42)
Emotion functioning	84 (20)	85 (13)
Cognitive functioning	82 (19)	72 (24)
Social functioning *	83 (21)	67 (37)
Fatigue *	29 (27)	47 (32)
Nausea/Vomiting	4.6 (11)	3.9 (10)
Pain*	19 (25)	45 (30)
Dyspnea	18 (26)	23 (25)
Insomnia	28 (30)	23 (29)
Appetite loss	13 (25)	21 (22)
Constipation	16 (25)	31 (42)
Diarrhea	8.8 (18)	5.1 (13)
Financial difficulties	12 (20)	13 (22)
QLQ-PR25		
Sexual activity	1.5 (4.8)	0.00 (0.00)
Sexual functioning	-	-
Urinary symptoms	24 (17)	33 (25)
Bowel symptoms	8.6 (11)	9.0 (11)
Hormonal treatment-related symptoms	11 (10)	22 (9.6)
Incontinence aid	17 (27) [n = 26]	50 (46) [n = 6]

ARSI = androgen receptor signaling inhibitors; EQ-5D-5L = EuroQoL five-dimension five-level questionnaire; EQVAS = EuroQoL visual analogue scale; HRQoL = health-related quality of life; QLQ-C30 = Quality of Life Questionnaire Core 30-item; QLQ-PR25 = Quality of Life Questionnaire Prostate Cancer 25-item.

*The mean difference between the groups exceeded the reference MID, suggesting potential clinical relevance.

Table s7 - HRQoL of patients with metastatic or non-metastatic prostate cancer

HRQoL	Non-metastatic prostate cancer patients (n = 18)	Metastatic prostate cancer patients (n = 82)
EQ-5D-5L		
EQ-5D index score	0.76 (0.45)	0.70 (0.40)
EQ VAS	77 (19)	74 (18)
QLQ-C30		
Global health status	72 (21)	69 (21)
Physical functioning *	78 (26)	71 (30)
Role functioning *	86 (28)	75 (33)
Emotion functioning	85 (17)	84 (19)
Cognitive functioning	84 (16)	80 (21)
Social functioning *	94 (12)	78 (26)
Fatigue	28 (21)	32 (29)
Nausea/Vomit	2.8 (6.4)	4.9 (12)
Pain	21 (28)	22 (27)
Dyspnea	11 (26)	20 (26)
Insomnia	24 (30)	28 (30)
Appetite loss	7.4 (14)	15 (26)
Constipation	20 (31)	17 (28)
Diarrhea	5.6 (13)	8.9 (18)
Financial difficulties	3.7 (11)	13 (21)
QLQ-PR25		
Sexual activity	3.7 (7.1)	0.81 (3.6)
Sexual functioning	-	-
Urinary symptoms	24 (15)	25 (19)
Bowel symptoms	7.4 (8.0)	8.9 (11)
Hormonal treatment-related symptoms	10 (8.1)	13 (11)
Incontinence aid	19 (18) [n=7]	24 (37) [n=25]

EQ-5D-5L = EuroQoL five-dimension five-level questionnaire; EQVAS = EuroQoL visual analogue scale; QLQ-C30 = Quality of Life Questionnaire Core 30; QLQ-PR25 = Quality of Life Questionnaire Prostate Cancer 25

*The mean difference between the groups exceeded the reference MID, suggesting potential clinical relevance.

Table s8 - Associated factors for EQ-5D-5L index score of all ARSI users

Variable	Univariable analysis				Multivariable analysis			
	Estimate (SE)	95% lower CI	95% upper CI	*p* value	Estimate (SE)	95% lower CI	95% upper CI	*p* value
Age (yr)								
<60	Ref				Ref			
60-69	-0.04 (0.22)	-0.47	0.39	0.86	0.08 (0.20)	-0.32	0.48	0.69
70-79	-0.20 (0.21)	-0.62	0.22	0.35	-0.05 (0.19)	-0.42	0.32	0.79
>80	-0.24 (0.22)	-0.67	0.19	0.27	0.05 (0.19)	-0.33	0.43	0.81
Disease duration (yr)	-0.004 (0.01)	-0.03	0.02	0.74	-0.004 (0.02)	-0.03	0.03	0.81
ARSI duration (month)	-0.003 (0.003)	-0.008	0.002	0.23	-0.003 (0.002)	-0.008	0.002	0.28
PSA at index date (ng/mL)	-0.004 (0.002)	-0.007	-0.001	0.02	-0.003 (0.001)	-0.005	-0.0001	0.04
Wheelchair using	-0.75 (0.09)	-0.92	-0.58	<0.001	-0.64 (0.11)	-0.86	-0.42	<0.001
With diaper	-0.24 (0.08)	-0.41	-0.07	0.01	-0.05 (0.08)	-0.22	0.11	0.52
Tumor metastasis	-0.07 (0.11)	-0.28	0.14	0.53	-0.12 (0.13)	-0.38	0.13	0.33

ADT = androgen deprivation therapy; ARSI = androgen receptor signaling inhibitor; BMI = body mass index; CI = confidence interval; EQ-5D-5L = EuroQoL five-dimension five-level questionnaire; LHRH = luteinizing-hormone releasing hormone; PSA = prostate-specific antigen; SE = standard error.

Variables used in exploration multivariable analysis were based on past research experience [4-7].

Section 3 Supplementary data on mapping

Table s9 -The correlation between EQ-5D-5L index score and QLQ-C30/PR25 subscales

QLQ-C30 and QLQ-PR25 subscales		Spearman correlation coefficient
QLQ-C30		
Qol	Global health status	0.56
PF	Physical functioning	0.78
RF	Role functioning	0.75
EF	Emotional functioning	0.32
CF	Cognitive functioning	0.45
SF	Social functioning	0.56
FA	Fatigue	-0.64
NV	Nausea/vomiting	-0.19
PA	Pain	-0.61
DY	Dyspnea	-0.50
SL	Insomnia	-0.32
AP	Appetite loss	-0.48
CO	Constipation	-0.35
DI	Diarrhea	-0.28
FI	Financial difficulties	-0.29
QLQ-PR25		
SA	Sexual activity	0.17
US	Urinary symptoms	-0.35
BS	Bowel symptoms	-0.49
HS	Hormone treatment-related symptoms	-0.31
IA	Incontinence aid	-0.24

EQ-5D-5L = EuroQoL five-dimension five-level questionnaire; QLQ-C30 = Quality of Life Questionnaire Core 30-item; QLQ-PR25 = Quality of Life Questionnaire Prostate Cancer 25-item.

Table s10 - Intercorrelation between the QLQ-C30 and QLQ-PR25 subscales

	Qol	PF	RF	EF	CF	SF	FA	NV	PA	DY	SL	AP	CO	DI	FI	SA	US	BS	HS	IA
Qol	1.00																			
PF	0.57	1.00																		
RF	0.51	0.72	1.00																	
EF	0.47	0.29	0.38	1.00																
CF	0.46	0.52	0.46	0.60	1.00															
SF	0.51	0.53	0.60	0.50	0.53	1.00														
FA	-0.46	-0.71	-0.72	-0.47	-0.58	-0.58	1.00													
NV	-0.30	-0.23	-0.26	-0.39	-0.30	-0.29	0.40	1.00												
PA	-0.46	-0.60	-0.59	0.44	-0.36	-0.51	0.63	0.29	1.00											
DY	-0.32	-0.50	-0.58	-0.33	-0.35	-0.38	0.57	0.34	0.49	1.00										
SL	-0.24	-0.27	-0.38	-0.40	-0.29	-0.32	0.32	0.27	0.40	0.40	1.00									
AP	-0.48	-0.46	-0.45	-0.50	-0.45	-0.45	0.52	0.29	0.47	0.31	0.28	1.00								
CO	-0.23	-0.43	-0.33	-0.27	-0.31	-0.31	0.39	0.07	0.36	0.27	0.32	0.29	1.00							
DI	-0.23	-0.24	-0.28	-0.34	-0.21	-0.15	0.32	0.30	0.31	0.42	0.23	0.19	0.07	1.00						
FI	-0.44	-0.28	-0.39	-0.52	-0.37	-0.62	0.40	0.27	0.38	0.25	0.20	0.35	0.15	0.11	1.00					
SA	0.12	0.11	0.04	-0.09	-0.10	0.07	-0.01	-0.03	-0.11	0.02	-0.03	-0.05	0.03	0.10	0.04	1.00				
US	-0.41	-0.42	-0.39	-0.44	-0.37	-0.51	0.42	0.15	0.34	0.29	0.36	0.34	0.20	0.19	0.33	-0.01	1.00			
BS	-0.42	-0.49	-0.53	-0.36	-0.35	-0.55	0.53	0.20	0.43	0.39	0.29	0.30	0.51	0.34	0.37	0.08	0.50	1.00		
HS	-0.37	-0.32	-0.36	-0.38	-0.34	-0.41	0.42	0.29	0.45	0.37	0.27	0.16	0.14	0.20	0.39	0.05	0.43	0.34	1.00	
IA	-0.54	-0.45	-0.41	-0.52	-0.59	-0.55	0.43	0.11	0.39	0.06	0.37	0.30	0.23	-0.04	0.47	0.00	0.65	0.36	0.52	1.00

AP = appetite loss; BS = bowel symptoms; CF = cognitive functioning; CO = constipation; DI = diarrhea; DY = dyspnea; EF = emotional functioning; FA = fatigue; FI = financial difficulties; HS = hormone treatment-related symptoms; IA = incontinence aid; NV = nausea/vomiting; PA = pain; PF = physical functioning; QLQ-C30 = Quality of Life Questionnaire Core 30-item; QLQ-PR25 = Quality of Life Questionnaire Prostate Cancer 25-item; Qol = The QLQ-C30 global health status score; RF = role functioning; SA = sexual activity; SF = social functioning; SL = insomnia; US = urinary symptoms.

Intercorrelations within the subscales of QLQ-C30 and QLQ-PR25 were assessed to gauge the potential strength of collinearity in subsequent regression analyses. The strength of coefficient was supposed to be moderate, then regression could be conducted. If the coefficient equaled or exceeded 0.6, the associated items were considered interaction terms.

Based on the results of intercorrelation between the QLQ-C30 and QLQ-PR25 subscales, the ***selected interaction variables*** were:
(1) physical functioning × role functioning, (2) QLQ-C30 global health status × physical functioning, (3) emotion functioning × cognitive functioning, and (4) role functioning × social functioning.

Table s11 - Model performance and evaluation metrics from OLS regression analysis

Regression models	Adjusted R^2	MAE	RMSE	AIC	BIC	MAE Rank	RMSE Rank	AIC Rank	BIC Rank	Average Rank
OLS_Model 1	0.81	0.13	0.18	-237	-336	3	2	4	6	6
OLS_Model 2	0.90	0.09	0.13	-302	-399	2	1	3	5	5
OLS_Model 3	0.91	0.087	0.13	-303	-402	1	1	2	3	3
OLS_Model 4	0.90	0.09	0.13	-304	-403	2	1	1	2	2
OLS_Model 5	0.81	0.13	0.18	-237	-336	3	2	4	6	6
OLS_Model 6	0.90	0.09	0.13	-302	-400	2	1	3	4	4
OLS_Model 7	0.91	0.087	0.13	-303	-402	1	1	2	3	3
OLS_Model 8	0.90	0.09	0.13	-304	-404	2	1	1	1	1

AIC = Akaike information criterion; BIC = Bayesian information criterion, OLS = ordinary least squares regression, RMSE = root of mean squared error.

OLS mapping equation of the best-fit model (average rank = 1):

Model 8 (with the variable "age"): Predicted EQ-5D index score = 0.21 + 0.019 × Physical functioning - 0.0084 × Diarrhea - 0.00010 × Physical functioning^2 - 0.000055 × Pain^2 + 0.00019 × Diarrhea^2 + 0.000017 × (Role functioning × Social functioning) - 0.0042 × Age

Table s12 - Model performance and evaluation metrics from Tobit regression analysis

Regression models	Log-likelihood	MAE	RMSE	AIC	BIC	MAE Rank	RMSE Rank	AIC Rank	BIC Rank	Average Rank
Tobit_Model 1	0.99	0.11	0.15	40	95	5	4	7	2	6
Tobit_Model 2	26	0.075	0.10	24	123	3	2	4	6	4
Tobit_Model 3	30	0.073	0.097	24	133	1	1	4	7	3
Tobit_Model 4	30	0.074	0.097	26	138	2	1	5	8	5
Tobit_Model 5	0.16	0.11	0.15	34	78	5	4	6	1	5
Tobit_Model 6	22	0.08	0.11	18	98	4	3	3	3	3
Tobit_Model 7	27	0.073	0.10	15	107	1	2	1	4	1
Tobit_Model 8	28	0.073	0.10	16	110	1	2	2	5	2

AIC = Akaike information criterion; BIC = Bayesian information criterion; MAE = mean absolute error; RMSE = root of mean squared error.

Unlike OLS, the predicted EQ-5D values from the Tobit model cannot be manually calculated using regression coefficients alone due to the censored nature of the model. The final predicted values were derived using the built-in algorithm in SAS PROC QLIM, which computes the conditional expectation based on the estimated latent variable, censoring threshold, and standard deviation.

Note:

1. Although the limited sample size restricted precise estimation of individual-level prediction errors, the developed mapping algorithms may still be applicable for individual use. However, external validation using larger and more diverse samples is necessary to improve predictive accuracy and generalizability across different patient populations.
2. The underlying code and data used to generate the models are available upon reasonable request to the corresponding author, promoting study reproducibility and transparency.

Table s13 - Distribution of observed and predicted EQ-5D-5L index scores and cross-validation of the mapping models

	EQ-5D-5L utility						5-fold cross-validation	
	Mean (SD)	Min	P25	P50	P75	Max	MAE	RMSE
Observed	0.71 (0.41)	-0.67	0.65	0.87	1.00	1.00	-	-
OLS_model 1	0.71 (0.37)	-0.38	0.57	0.81	1.00	1.11	0.13	0.17
OLS_model 2	0.71 (0.39)	-0.67	0.66	0.87	0.95	1.01	0.09	0.12
OLS_model 3	0.71 (0.39)	-0.69	0.66	0.86	0.95	1.04	0.09	0.12
OLS_model 4	0.71 (0.39)	-0.78	0.66	0.86	0.93	1.02	0.09	0.12
OLS_model 5	0.71 (0.37)	-0.38	0.57	0.81	1.00	1.11	0.13	0.17
OLS_model 6	0.71 (0.39)	-0.67	0.66	0.87	0.95	1.01	0.09	0.12
OLS_model 7	0.71 (0.39)	-0.68	0.67	0.85	0.96	1.01	0.09	0.12
OLS_model 8	0.71 (0.39)	-0.78	0.66	0.86	0.93	1.02	0.09	0.12
Tobit_model 1	0.72 (0.38)	-0.41	0.60	0.90	1.00	1.00	0.11	0.15
Tobit_model 2	0.73 (0.41)	-0.67	0.66	0.90	1.00	1.00	0.08	0.10
Tobit_model 3	0.73 (0.41)	-0.74	0.67	0.88	1.00	1.00	0.07	0.10
Tobit_model 4	0.73 (0.41)	-0.74	0.67	0.88	1.00	1.00	0.07	0.10
Tobit_model 5	0.72 (0.38)	-0.45	0.59	0.88	1.00	1.00	0.11	0.15
Tobit_model 6	0.73 (0.41)	-0.63	0.66	0.90	1.00	1.00	0.08	0.11
Tobit_model 7	0.73 (0.41)	-0.71	0.66	0.90	1.00	1.00	0.07	0.10
Tobit_model 8	0.73 (0.41)	-0.71	0.66	0.89	1.00	1.00	0.07	0.10

MAE = mean absolute error; Max = Maximum; Min = minimum; OLS = ordinary least squares; P25 = 25th percentile; P50 = 50th percentile; P75 = 75th percentile; RMSE = root of mean squared error.

Table s14 - Comparison of model fit between OLS models (Partial F-test)

Comparison	ΔDF	F value	*p* value	Interpretation
Model 1 vs Model 5	4	0.44	0.78	Adding QLQ-PR25 subscales did not improve overall model fit and explanatory power.
Model 2 vs Model 6	7*	0.89	0.52	
Model 3 vs Model 7	7*	0.57	0.78	
Model 4 vs Model 8	7*	0.53	0.81	

* Due to the limited variability of the subscale sexual activity (many patients scored 0), the squared term of sexual activity caused estimation issues and was excluded from both models.

This adjustment did not alter the model interpretation and allowed valid F-tests for model comparison.

Table s15 - Comparison of model fit between Tobit models (Likelihood Ratio Test)

Comparison	ΔDF	LRT	*p* value	Interpretation
Model 1 vs Model 5	4	1.7	0.80	Adding QLQ-PR25 subscales did not improve overall model fit and explanatory power.
Model 2 vs Model 6	8	7.3	0.51	
Model 3 vs Model 7	8	5.3	0.73	
Model 4 vs Model 8	8	4.6	0.80	

LFT = Likelihood Ratio Test.

References

[1] Krejcie RV, Morgan DW. Determining Sample Size for Research Activities. *Educ Psychol Meas*. 1970;30(3):607-610.

[2] Henry EB, Devlin N, van Hout BA, Rand K. Estimation of an Instrument-Defined Minimally Important Difference in EQ-5D-5L Index Scores Based on Scoring Algorithms Derived Using the EQ-VT Version 2 Valuation Protocols. *Value Health*. 2020;23(7):936-944.

[3] Gamper EM, Stopp E, von der Lohe J, et al. Minimally important differences for the EORTC QLQ-C30 in prostate cancer clinical trials. *BMC Cancer*. 2021;21(1):1083.

[4] Zheng Z, Hu W, Li Y, et al. Health-related quality of life among prostate cancer survivors with metastatic disease and non-metastatic disease and men without a cancer history in the USA. *J Cancer Surviv*. 2025;19(3):871-883.

[5] Sun N, Gu Y. Exploring the Hidden Struggles: A Qualitative Insight into Urinary Incontinence Among Prostate Cancer Survivors Post-Surgery. *Patient Prefer Adherence*. 2024;18:1047-1058.

[6] Feyzioğlu Ö, Demirel Ş, Özdemir AE, Öztürk Ö, Açan Hİ. Effects of Androgen Deprivation Therapy Duration on Health-Related Quality of Life, Physical Activity, Anxiety and Depression Levels in Patients with Intermediate- and High-Risk Prostate Cancer. *Arch Health Sci Res*. 2024;11(1):14-19.

[7] Cormier L, Eifler J, Bladou F, et al. Impact of prostate cancer screening on health-related quality of life in at-risk families. *Urology*. 2002;59(6):901-906.
